# The Carbohydrate Metabolism of *Lactiplantibacillus plantarum*

**DOI:** 10.3390/ijms222413452

**Published:** 2021-12-15

**Authors:** Yanhua Cui, Meihong Wang, Yankun Zheng, Kai Miao, Xiaojun Qu

**Affiliations:** 1 Department of Food Nutrition and Health, School of Medicine and Health, Harbin Institute of Technology, Harbin 150001, China; wangmh644@163.com (M.W.); zhengyankun0301@163.com (Y.Z.); 17862516319@163.com (K.M.); 2 Institute of Microbiology, Heilongjiang Academy of Sciences, Harbin 150010, China; qvxiaojun@163.com

**Keywords:** *Lactiplantibacillus plantarum*, carbohydrate metabolism, two-component system

## Abstract

*Lactiplantibacillus plantarum* has a strong carbohydrate utilization ability. This characteristic plays an important role in its gastrointestinal tract colonization and probiotic effects. *L. plantarum* LP-F1 presents a high carbohydrate utilization capacity. The genome analysis of 165 *L. plantarum* strains indicated the species has a plenty of carbohydrate metabolism genes, presenting a strain specificity. Furthermore, two-component systems (TCSs) analysis revealed that the species has more TCSs than other lactic acid bacteria, and the distribution of TCS also shows the strain specificity. In order to clarify the sugar metabolism mechanism under different carbohydrate fermentation conditions, the expressions of 27 carbohydrate metabolism genes, catabolite control protein A (CcpA) gene *ccpA,* and TCSs genes were analyzed by quantitative real-time PCR technology. The correlation analysis between the expressions of regulatory genes and sugar metabolism genes showed that some regulatory genes were correlated with most of the sugar metabolism genes, suggesting that some TCSs might be involved in the regulation of sugar metabolism.

## 1. Introduction

*Lactiplantibacillus plantarum* is a highly versatile and flexible species that distributes in a wide variety of habitats such as vegetables, dairy product, meat, as well as grass silage [1,2,3]. Furthermore, it can also be found in the human body as a natural inhabitant, for example in the saliva [4] and gastrointestinal tract of humans [5]. Some studies have demonstrated that *L. plantarum* possesses some beneficial functions for host, such as maintaining the balance of gastrointestinal flora, improving self-immunity of host, promoting effective absorption of nutrients, reducing cholesterol content, and alleviating lactose intolerance [6].

*L. plantarum* has been widely used in the production of various fermented foods as a starter culture, giving to the flavor, texture, and organoleptic properties of products. It could produce some bioactive substances such as exopolysaccharides, γ-aminobutyric acid, folic acid, and riboflavin, which offer functional properties of the fermented foods with the species [7,8,9]. Furthermore, it can be used as a food preservative in food processing and preservation via the production of bacteriocin and organic acid [6].

Sugars are important energy and structural substances in bacteria. Compared with most lactic acid bacteria (LAB), *L. plantarum* presents a stronger carbohydrate utilization capability, which contributes to a broad adaptability in various environments with different carbohydrates [10,11,12,13]. The species could not only use cellobiose, mannose, D-ribose, and L-fucose, but also ferment prebiotics fructooligosaccharides (FOS) and galactooligosaccharide (GOS) [13,14]. The organism contains a comprehensive carbohydrate utilization system composed of a plenty of sugar uptake and metabolism related genes, which endow its strong carbohydrate utilization ability [4]. The number of these sugar metabolism related genes is far more than those found in other lactic acid bacterial genomes [4]. Different *L. plantarum* strains show various carbohydrate utilization profiles [10,12,13].

Researchers pay attention to the unique characteristics and strong adaptive ability of *L. plantarum*. Up to July 2021, 612 *L. plantarum* strains have been sequenced at the genome level. Among these strains, the complete genome sequences of 149 strains have been finished, and 11, 254, and 198 strains are under chromosome, contig, and scaffold stages, respectively. Compared with other LAB species genomes, *L. plantarum* genomes have larger sizes, ranging from 2.91 to 3.70 Mb. The large size of genome might have a close relationship with the ability of this bacterium to inhabit diverse environmental niches [15]. Furthermore, extensive post-genomic analyses have been performed on *L. plantarum* species in recent years [12,16,17,18].

Two-component systems (TCSs) are important signal transduction systems in most Gram-positive and Gram-negative bacteria, regulating diverse physiological processes and metabolisms, such as bacteriocin biosynthesis, proteolytic ability, acid tolerance ability, antibiotic resistance, and virulence factor production [19,20,21,22]. TCS is composed of a sensor histidine protein kinase (HPK) and a response regulatory protein (RR) [21]. HPK is responsible for receiving the environmental signals, and RR is involved in the regulation of the target genes [21].

Compared with other LAB species, more TCSs are found in the *L. plantarum* genome. Generally, *L. plantarum* strains contain 13–14 TCSs, while *Lactobacillus delbrueckii* subsp. *bulgaricus* strains have 5–7 TCSs, *S. thermophilus* strains possess 6–8 TCSs [4,23,24]. It is speculated that a large number of TCSs facilitate and improve the survival ability and adaptability of the species in various environments. It is worth noting that five TCSs in *L. plantarum* are belong to components of quorum sensing systems by in silico analysis [25].

A TCS consisting of an HPK PlnB and two RRs PlnC and PlnD has been identified to be responsible for the plantaricin biosynthesis in *L. plantarum* [26]. Furthermore, an *agr*-like TCS LamCA has been demonstrated to be involved in the regulation of bacterial adherence via a cyclic peptide as a signal in *L. plantarum* WCFS1 [27]. In addition, the LamK (HPK10) and LamR (RR10) are highly homologous to the LamC and LamA, respectively, and their amino acid sequences identities are 55% and 70% [28]. The cooperative effect of LamA and LamR has been revealed by means of a series of experiments, including transcription analysis of the *lamKR* operon and the *lamBDCA* operon, liquid chromatography-mass spectrometry analysis of production of the LamD558 autoinducing peptide, and mutant analysis. The *ΔlamA* and *ΔlamAΔlamR* mutants (the *lamA* and *lamR* double-knockout mutant) reduced adherence to glass surfaces, the latter with more significant reduced adherence ability to glass surfaces than the former [28]. The LamA and LamR regulated the expressions of cell wall polysaccharide synthesis genes, stress response-related genes, and cell wall protein-encoding genes. Although the sequences of some TCSs in the *L. plantarum* show similarity with those of known function TCSs in other species, the functions of these TCSs are not clear in the species.

In our previous study, *L. plantarum* LP-F1 was isolated from fermented milk, presenting an outstanding fermentation ability and bioactive substance biosynthesis capability [29]. In the present study, the genome of *L. plantarum* LP-F1 has been sequenced. The carbohydrate metabolism related genes of 165 *L. plantarum* strains containing LP-F1 strain have been analyzed in order to reveal the differences of different strains of same species in the carbohydrate metabolism at molecular levels. Furthermore, the expressions of key sugar metabolism genes of LP-F1 strain under the conditions with prebiotics FOS and inulin were determined for clarifying the relationship of different sugar metabolism pathways. As important signal transduction and regulation systems, the expressions of catabolite control protein A (CcpA) and TCS genes were analyzed during the carbohydrate metabolism in order to reveal their roles in bacterial carbohydrate utilization. Moreover, correlation analysis between the expressions of regulated genes and different sugar metabolism related genes was performed in order to establish their relationships and guild a basement for the regulation in sugar metabolism in application in the future.

## 2. Results

### 2.1. The Sugar Fermentation Capacity of L. plantarum Strains

The sugar fermentation capacities of *L. plantarum* strains LP-F1, LP-E1, LP-A4, LP-I5, LP-1, and LP-4 isolated from fermented milk samples were evaluated (Supplementary Table S1). All tested strains could utilize D-glucose, D-galactose, D-fructose, D-lactose, D-sucrose, D-maltose, D-mannose, D-salicin, D-xylose, D-ribose, D-mannitol, and D-sorbitol, while they could not ferment galactitol, L-fucose, L-rhamnose, and D-melibiose. In addition, these strains present different abilities to ferment D-mannitol, D-sorbitol, L-arabinose, L-sorbose, and D-melezitose. It is worth noting that these strains could metabolize FOS and inulin. These strains present different sugar fermentation capacities. The results are consistent with the previous studies [10,12,13]. It is found that *L. plantarum* strains LP-F1 presented a strong sugar fermentation ability among these tested strains, especially in the FOS and inulin metabolism. The capability to utilize carbohydrates depends on the presence of a functional transport system and intracellular metabolic pathways. The genome of LP-F1 was sequenced in order to reveal its carbohydrate metabolism related genes.

### 2.2. Genomic Analysis of L. plantarum Strain LP-F1

**Figure 1:**
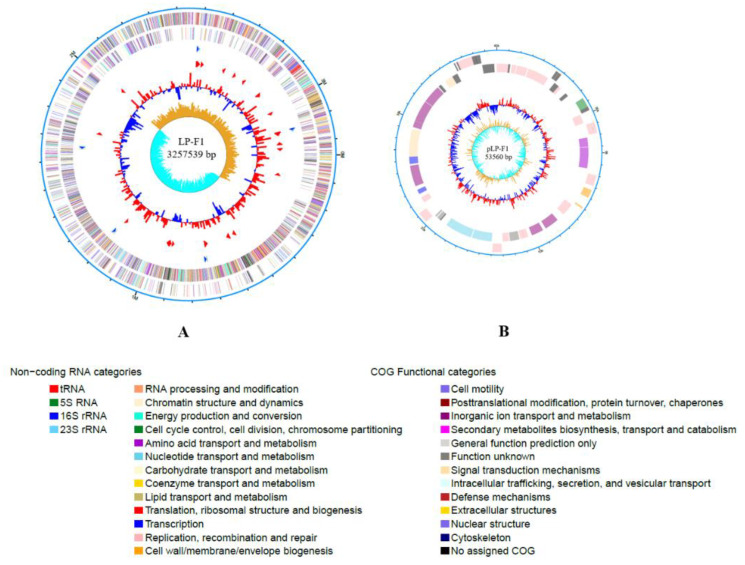
Circular genome map of the *L. plantarum* LP-F1 chromosome and plasmid. (**A**) Chromosome. (**B**) Plasmid pLP-F1.

*L. plantarum* LP-F1 genome was sequenced by a combination of Pacbio RS and Illumina sequencing technology. LP-F1 contains one chromosome (3, 257, 539 bp), and one plasmid pLP-F1 (53, 560 bp), both of which are circular in molecular type, as shown in Figure 1. 15 *L. plantarum* strains genomes available in NCBI databases were used in a phylogenetic analysis with LP-F1 (Supplementary Table S2). It is found that the length of *L. plantarum* genomes ranged from 2.99 to 3.79 Mb, the G + C% contents were 43.3–46.5%. Compared with other *L. plantarum* strains, the genome size of LP-F1 is medium.

**Figure 2:**
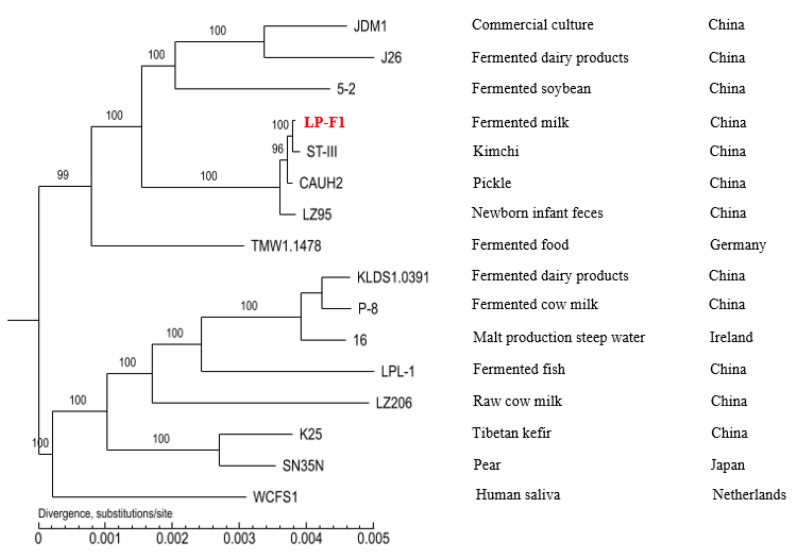
Phylogenetic trees of 16 *L. plantarum* strains. In order to highlight the strain LP-F1 in this study, the strain name LP-F1 is shown in red.

In this study, the phylogenetic analysis of 16 *L. plantarum* strains isolated from various environments was carried out based on the conserved proteins detected in all tested genome sequences (Figure 2). These strains were obtained from different environments, such as fermented dairy products, fermented fish, fermented vegetables, raw milk, fruit, infant feces, and human saliva [4,30,31,32,33,34,35,36,37].

Seven strains from China are clustered into the same branch. However, K25 and SN35N are clustered together, which were from China and Japan, respectively [32,34]. Strain 16 from Ireland has a closer relationship with KLDS1.0391 and P-8 than those of other strains from China [30,31,35]. Therefore, it could be concluded that the phylogenetic reconstruction based on the sequenced genomes of strains was not obviously associated with their geographic distributions.

Furthermore, strains from different isolation sources were also clustered together. For example, strains LP-F1, ST-III, and CAUH2 are clustered on the same branch, while the former from fermented milk and the latter two from fermented vegetables [36,37]. The results indicated that the survival environments of strains are not resulting in the differences in their genomes. The results were consistent with those of previous studies [18,38].

### 2.3. Carbohydrate Metabolism Systems Analysis of LP-F1 Based on the Genome

**Figure 3:**
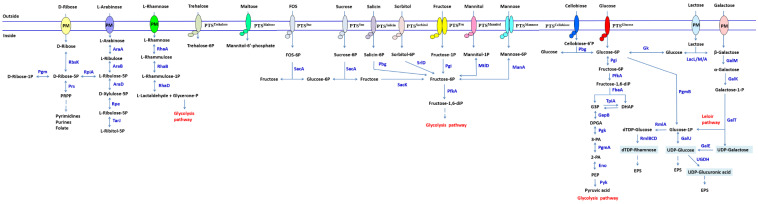
Sugar metabolism and central carbon pathways in *L. plantarum* LP-F1. 2-PA, 2-phosphoglyceric acid; 3-PA, 3-phosphoglyceric acid; AraA, L-arabinose isomerase; AraB, L-ribulokinase; AraD, L-ribulose 5-phosphate 4-epimerase; DHAP, dihydroxyacetone phosphate; DPGA, 1, 3-diphosphoglyceric acid; Eno, enolase; EPS, exopolysaccharide; FbaA, fructose bisphosphate aldolase; G3P, glyceralodehyde-3-phosphate; GalE, UDP-galactose 4-epimerase; GalK, galactokinase; GalM, galactose epimerase; GalT, UDP-glucose: alpha-D-galactose-1-phosphate uridylyltransferase; GalU, UDP-glucose pyrophosphorylase; GapB, glyceraldehyde-3-phosphate dehydrogenase; Gk, glucokinase; LacA, beta-galactosidase; LacL, beta-galactosidase large subunit; LacM, beta-galactosidase small subunit; ManA, mannose-6-phosphate isomerase; MtlD, mannitol-1-phosphate 5-dehydrogenase; Pbg, 6-phosphate -β-glucosidase; PEP, phosphoenolpyruvate; PfkA, 6-phosphofructokinase; Pgi, glucose-6-phosphate isomerase; Pgk, phosphoglycerate kinase; Pgm, beta-phosphoglucomutase; PgmA, phosphoglycerate mutase; PRPP, 5-phosphoribosyldiphosphate; Prs, ribose-phosphate pyrophosphokinase; Pyk, pyruvate kinase; RbsK, ribokinase; RhaA, L-rhamnose isomerase; RhaB, rhamnulokinase; RhaD, rhamnulose-1-phosphate aldolase; RmlA, glucose-1-phosphate thymidylyltransferase; RmlB, dTDP-glucose 4, 6-dehydratase; RmlC, dTDP-4-dehydrorhamnose 3,5-epimerase; RmlD, dTDP-4-keto-L-rhamnose reductase; Rpe, ribulose-phosphate 3-epimerase; RpiA, ribose-5-phosphate epimerase; SacA, sucrose-6-phosphate hydrolase; SacK, fructokinase; SrlD1/SrlD2, sorbitol-6-phosphate 2-dehydrogenase;TarJ, ribitol-5-phosphate 2-dehydrogenase; TpiA, triosephosphate isomerase; UGDH, UDP-glucose 6-dehydrogenase. The important pathways are shown in red.

**Figure 4:**
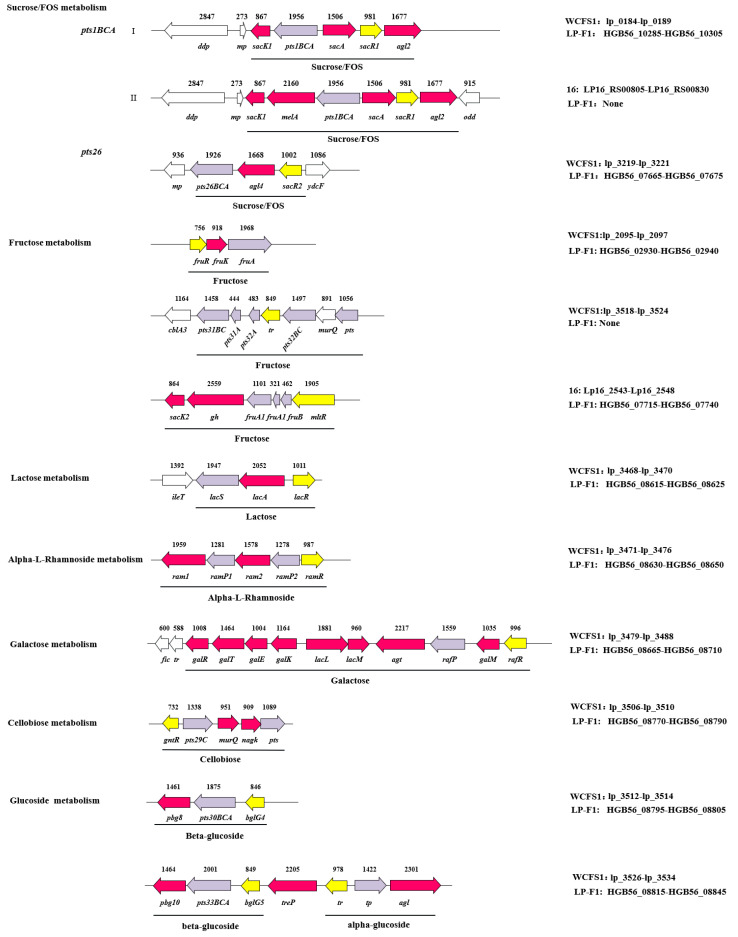

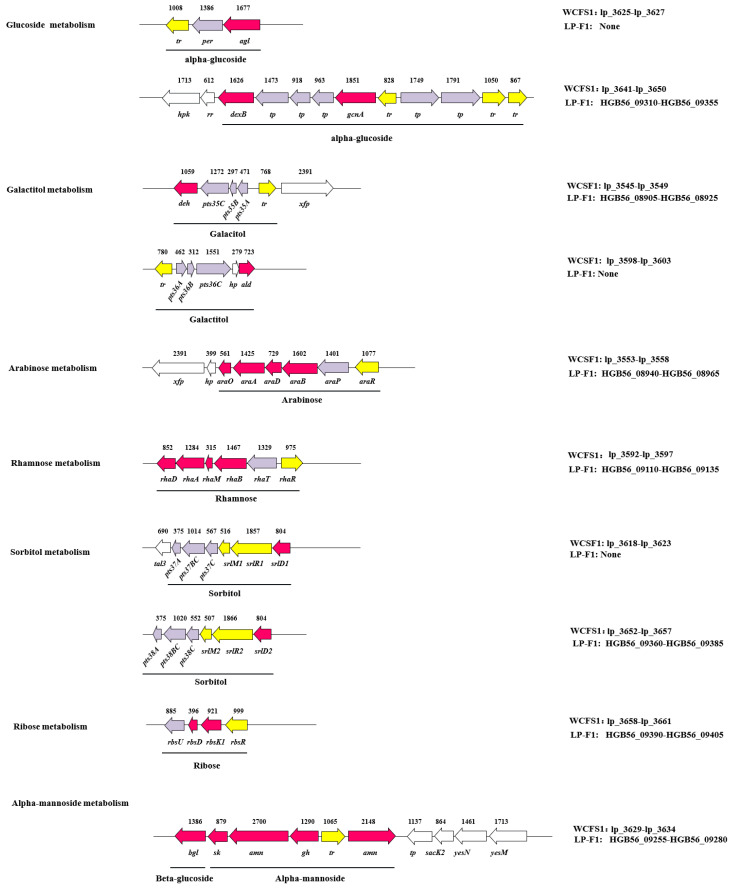
The distribution of carbohydrate metabolism genes clusters in the *L. plantarum* LP-F1.

*L. plantarum* is a heterofermentative lactic acid bacterium, which possesses both glycolysis and phosphoketolase pathways. The genes encoding enzymes responsible for these two pathways have been found on the strain LP-F1’s chromosome. Bioinformatics analysis revealed that LP-F1 strain possesses a plenty of carbohydrate utilization genes (Figure 3 and Figure 4, Supplementary Table S3-1-1). There are 130 enzymes related to carbohydrate metabolism in *L. plantarum* LP-F1 based on the CAZy analysis, which belong to auxiliary activities (AAs, 9), carbohydrate-binding modules (CBMs, 17), carbohydrate esterases (CEs, 16), glycoside hydrolases (GHs, 53), and glycosyl transferases (GTs, 35) (Supplementary Table S3-1-2). Some enzymes belong to two classes.

#### 2.3.1. Monosaccharide and Disaccharide

##### Glucose

*L. plantarum* transports glucose through PTS11ABC encoded by *pts11A* and *pts11BC*, and glucose enters cells in the form of 6-phosphate-glucose. The latter is transformed into 6-phosphate-fructose by 6-phosphate-glucose isomerase (*pgi*) and metabolized by glycolysis pathway (Figure 3). The most genes encoding enzymes in the glycolysis pathway located in two operons HGB56_12590-HGB56_12605 and HGB56_01890-HGB56_01895 (Supplementary Table S3-1). The organization is similar to those in the other LAB. It was considered that this genetic linkage is beneficial for the regulation of expressions of these enzymes according to the change of the level and type of available sugars in the habitat [4]. Furthermore, two phosphopyruvate hydratases were found in the genome.

##### Lactose and Galactose

The lactose is fermented by the lactose gene cluster (HGB56_08615-HGB56_08625), encoding a lactose permease (*lacS*, HGB56_08615), a beta-galactosidase (*lacA*, HGB56_08620) and a regulator protein(*lacR*, HGB56_08625). Except few strains, for examples LZ206, KLDS1.0391, 16, and P-8, most strains have this gene cluster (Supplementary Table S3-2). The gene cluster was also found in the *Lactiplantibacillus argentoratensis*, *Lactiplantibacillus paraplantarum*, and *Lactiplantibacillus pentosus* (Supplementary Table S3-2). The lactose entered cell by the permease, and was catalyzed by beta-galactosidase, producing galactose and glucose.

The galactose is metabolized via Leloir pathway. A *gal* operon *galKETRM* is involved in encoding of enzymes of the Leloir pathway in *L. plantarum* LP-F1, consisting of a galactokinase gene *galK* (HGB56_08680), an UDP-galactose 4-epimerase gene *galE* (HGB56_08675), a galactose-1-phosphate uridylyltransferase gene *galT* (HGB56_08670), a repressor protein gene *galR* (HGB56_08665), a galactose mutarotase gene *galM* (HGB56_08705). Furthermore, the genes *lacL* (HGB56_08685) and *lacM* (HGB56_08690) encode large subunit and small subunit of beta-galactosidase, respectively. The gene HGB56_08695 encodes an alpha-galactosidase. The gene HGB56_08700 encodes an MFS transporter. The locus is present in most *L. plantarum* strains (Supplementary Table S3-3).

##### Fructose and Sucrose

The strain LP-F1 contained multiple phosphotransferase systems (PTSs) responsible for fructose transport, which were encoded by *fruA* (HGB56_02940), *fruA1-fruA2-fruB* (HGB56_07725, HGB56_07730, HGB56_07735), *pts9ABCD* (HGB56_11950, HGB56_11955, HGB56_11960) and *pts10AB* (HGB56_11975, HGB56_11980) genes, respectively (Supplementary Table S3-4). Fructose enters cells in the form of 6-phosphate-fructose through these PTSs, then 6-phosphate-fructose is transformed into 1, 6-2P-fructose at the action of phosphofructokinase encoded by *pfk* gene, and enters the glycolysis pathway (Figure 3).

The *sacPTS1* gene cluster is involved in sucrose metabolism in *L. plantarum*, encoding fructokinase (*sacK1*), sucrose PTS (*pts1BCA*), 6-phosphate sucrose hydrolase (*scrB*, also known as *sacA*), sucrose operon repressor protein (*srcR*, also known as *sacR1*) and α-glucosidase (*agl2*) (Figure 3, Supplementary Table S3-5). Furthermore, the gene cluster is also involved in FOS utilization [39].

##### Arabinose

A gene cluster (HGB56_08940-HGB56_08965) related to the L-arabinose metabolism was found in the strain LP-F1, encoding a arabinose O-acetyltransferase (HGB56_08940), L-arabinose isomerase (*araA*, HGB56_08945), L-ribulose 5-phosphate 4-epimerase (*araD*, HGB56_08950), L-ribulokinase (*araB*, HGB56_08955), arabinose transporter (*araP*, HGB56_08960), transcription regulator (*araR*, HGB56_08965, GntR family), and hypothetical protein (HGB56_08935). The strains WCFS1, LP-F1, JDM1, 5-2, SN35N, K25, ST-III, CAUH2, J26, and LZ95 possess the gene cluster (Supplementary Table S3-6). Some strains have not the gene cluster, for examples, LZ206, KLDS1.0391, 16, LPL-1, P-8, and TMW1.1478. Furthermore, the arabinose gene cluster has been found in a few *L. paraplantarum* and *L. pentosus* strains.

The previous study revealed the arabinose gene cluster indeed perfectly correlates with the growth ability on the medium containing L-arabinose via the analysis of the relationship between the fermentation experiment and genome sequences (i.e., the strain with the gene cluster could grow on L-arabinose, while the strain without the gene cluster does not grow on L-arabinose) [12,17].

##### Rhamnose

The rhamnose gene cluster *rhaDAMBTR* (HGB56_09110-HGB56_09135) was identified in the strain LP-F1, composed of a rhamnulose-1-phosphate aldolase gene (*rhaD*, HGB56_09110), a L-rhamnose isomerase gene (*rhaA*, HGB56_09115), a L-rhamnose mutarotase gene (*rhaM*, HGB56_09120), a rhamnulokinase gene (*rhaB*, HGB56_09125), a beta-rhamnose transporter gene (major facilitator superfamily, *rhaT*, HGB56_09130), and a transcription regulator gene (*rhaR*, HGB56_09135). It is worth noting the L-rhamnose isomerase gene is a pseudo gene in strain LP-F1, which probably influences the L-rhamnose utilization. The strains WCFS1, JDM1, 5-2, TMW 1.1478, LPL-1, LZ95, CAUH2, and ST-III contain *rhaDAMBTR* (Supplementary Table S3-7). However, the gene cluster is absent in some strains, such as LZ206, KLDS1.0391, 16, SN35N, K25, P-8, and J26. In addition, the rhamnose gene cluster is present in a few *L. pentosus*, *Latilactobacillus sakei*, *Ligilactobacillus salivarius*, *Pediococcus acidilactici*, and *Pediococcus pentosaceus* strains.

#### 2.3.2. Sugar Alcohol

##### Galactitol

The gene cassette (HGB56_08905- HGB56_08920) is involved in uptake and metabolism of galactitol, consisting of a three-component PTS gene (HGB56_08910, HGB56_08915, HGB56_08920) and a dehydrogenase gene (HGB56_08905). The galactitol gene cluster is conserved in most strains (Supplementary Table S3-8). Another gene cassette (lp_3599-lp_3603) encodes a PTS (lp_3599, lp_3600, lp_3601), a sugar-phosphate aldolase, and a hypothetical protein (lp_3602), which is also responsible for the galactitol utilization, and the gene cluster was only found in limited strains, such as WCFS1, JDM1, and LPL-1(Supplementary Table S3-8).

##### Sorbitol

The sorbitol gene cluster *sor1* (HGB56_09360-HGB56_09385) is present in the *L. plantarum* LP-F1, encoding a PTS, a sorbitol operon activator, a sorbitol operon transcription antiterminator, and a sorbitol-6-phosphate 2-dehydrogenase. The gene cluster is conserved in most tested *L. plantarum* strains except strain J26 only with a sorbitol-6-phosphate 2-dehydrogenase gene (Supplementary Table S3-9). In addition, *L. plantarum* WCFS1 has another sorbitol gene cluster (lp_3618-lp_3623), which is absent in the other *L. plantarum* strains. These two operons have same organization structures, and have about 58.68% nucleotide sequence identity.

#### 2.3.3. Oligosaccharides and Polysaccharides

The genome analysis showed that LP-F1 strain contained *sacPTS1* and *sacPTS26* gene clusters (Figure 3, Supplementary Table S3-5). Studies have confirmed that *sacPTS1* gene cluster is responsible for short chain FOS metabolism in *L. plantarum* WCSF1 [39]. The short chain FOS was transported into cells via sucrose PTS, then it was hydrolyzed at the catalysis of 6-phosphate sucrose hydrolase to produce 6-phosphate-glucose and fructose. Furthermore, another gene cluster *sacPTS26* (LPST_C2650 to LPST_C2652) was found in ST-III strain, and was also involved in the utilization of FOS, was composed of *pts26BCA*, *agl4* and *sacR2* [40]. The genomic analysis indicated that most *L. plantarum* strains harbor *sacPTS1* and *sacPTS26* gene clusters, which could contribute to the ability to ferment FOS (Supplementary Table S3-5). Interestingly, almost half of strains contain an additional alpha-galactosidase gene in the *sacPTS1* gene cluster, compared with that of *L. plantarum* WCSF1.

In *L. plantarum* P14 and P76, the *fosRABCDXE* gene cluster was found to be involved in inulin utilization, encoding a PTS (*fosABCD*), β-fructosidase (*fosE*), a transcription regulator (*fosR*) and unknown functional protein (*fosX*). In the FOS metabolism regulated by *sacPTS1* and *sacpPTS26* gene clusters, FOS are first transported to cells and then catabolized; while the inulin metabolism regulated by *fosRABCDXE* gene cluster is that inulin is hydrolyzed to sucrose and fructose by β-fructosidase outside cells and then into intracellular metabolism [10]. Bioinformatics analysis showed that *fosRABCDXE* gene cluster was not found in other *L. plantarum* strains including LP-F1, indicating that the gene cluster rarely appeared in *L. plantarum*. Therefore, it needs to be further studied how *L. plantarum*, which does not contain *fosRABCDXE* gene cluster but can utilize inulin, metabolizes inulin.

#### 2.3.4. The Difference of Different *L. plantarum* Strains in Carbohydrates Utilization Genes

The *L. plantarum* strains present a plenty of diversity in the carbohydrate utilization-related key genes profiles (Supplementary Tables S4-1 and S4-2). A few carbohydrate metabolism genes are conserved in the *L. plantarum*, such as ribose and rhamnose utilization-related genes. However, most carbohydrates utilization genes in *L. plantarum* strains are various. For examples, some *L. plantarum* strains contain two galactitol gene clusters, whereas most strains only have one galactitol gene cluster. Some *L. plantarum* strains lack arabinose metabolism gene cluster *araOADBPR* and rhamnose utilization gene cluster *rhaDAMBTR*. The strains B21, GR0128, NCIMB8826, NCU116, SRCM102022, SRCM103357, and WCFS1 contain all tested sugar fermentation gene clusters (Supplementary Tables S4-1 and S4-2). It was speculated that these strains have stronger sugar fermentation capabilities compared with other strains.

The carbohydrate metabolism genes in strains are key factors for the carbohydrate utilization capability. The genes will contribute to the sugar fermentation potentials of strains. These results indicated that *L. plantarum* strains present different carbohydrate utilization capabilities since they possess various carbohydrate utilization-related key genes profiles.

*L. plantarum* strains are isolated from different ecological niches, most of them from fermented vegetables, other strains from raw fruits and vegetables, raw milk, fermented milk, healthy infant fecal samples, wine, etc. [1,3,4,11,12,13,30,31,32,33,34,35,36,37,38]. Some strains from the same niches present the similar carbohydrate metabolism genes. For examples, *L. plantarum* ATG-K6 and ATG-K8 isolated from kimchi [41], these two strains possess the same gene clusters responsible for the utilization of sucrose, cellobiose, lactose, galactose, fructose, sorbitol, galactitol, arabinose, rhamnose, and ribose (Supplementary Tables S4-1 and S4-2). *L. plantarum* SRCM100438, SRCM100440, and SRCM100442 from infant feces samples, these three strains harbor the same carbohydrate utilization genes.

Furthermore, some strains from the same habitat also have different carbohydrate metabolism genes profiles, for example, strains from the fermented vegetables show obvious differences in the sugar fermentation genes (Supplementary Tables S4-1 and S4-2). In addition, some strains from different niches possess same carbohydrate metabolism genes profiles, such as *L. plantarum* LPL-1 and b-2 have same sugar fermentation genes, whereas they were isolated from fermented fish and pickle, respectively [42] (Supplementary Tables S4-1 and S4-2). The situation is also present in the other LAB with wide distribution, such as *Lacticaseibacillus casei*, *Lacticaseibacillus paracasei*, and *Lacticaseibacillus rhamnosus* [14]. Therefore, the sugar fermentation capability of strains might not depend on the isolation sources of strains.

What’s more, the related species of *L. plantarum*, *L. paraplantarum* and *L. pentosus*, belong to *Lactiplantibacillus* genus, some strains of them harbor some similar carbohydrate metabolism genes with *L. plantarum*, such as lactose, galactose, arabinose, rhamnose, ribose, sucrose, and sorbitol utilization genes (Supplementary Tables S4-1 and S4-2). Furthermore, similar cellobiose and rhamnoside metabolism genes are found in some *L. paraplantarum* strains, and alpha-glucosides and galactitol gene clusters are present in some *L. pentosus* strains. These results indicated that these species have a close relationship. It is worth noting that the genome information of *L. pentosus* and *L. paraplantarum* is limited. Up to August 2021, 53 *L. pentosus* strains and 13 *L. paraplantarum* strains have been sequenced at the genome level. Among these strains, the complete genome sequences of 5 *L. pentosus* strains have been finished, and 2, 29, and 17 *L. pentosus* strains are under chromosome, contig, and scaffold stages, respectively. The complete genomes sequences of 3 *L. paraplantarum* strains have been determined, 1, 5 and 4 *L. paraplantarum* strains are under chromosome, contig and scaffold stages, respectively.

Furthermore, compared with other LAB species, *L. plantarum* also presents obvious differences in the carbohydrate metabolism genes. For examples, the species *Lactic. casei*, *Lactic. paracasei*, and *Lactic. rhamnosus,* common LAB in fermented milk, ferment FOS via a *fosRABCDXE* operon, while most *L. plantarum* strains take *sacPTS1* and *sacPTS26* gene clusters to utilize FOS [14]. *Lactic. casei* and *Lactic. paracasei* metabolize galactose by means of Leloir and tagatose-6-phosphate pathways [14]. However, galactose is fermented via Leloir pathway in *L. plantarum.*

### 2.4. Two-Component Systems Analysis of LP-F1 Based on the Genome

**Figure 5:**
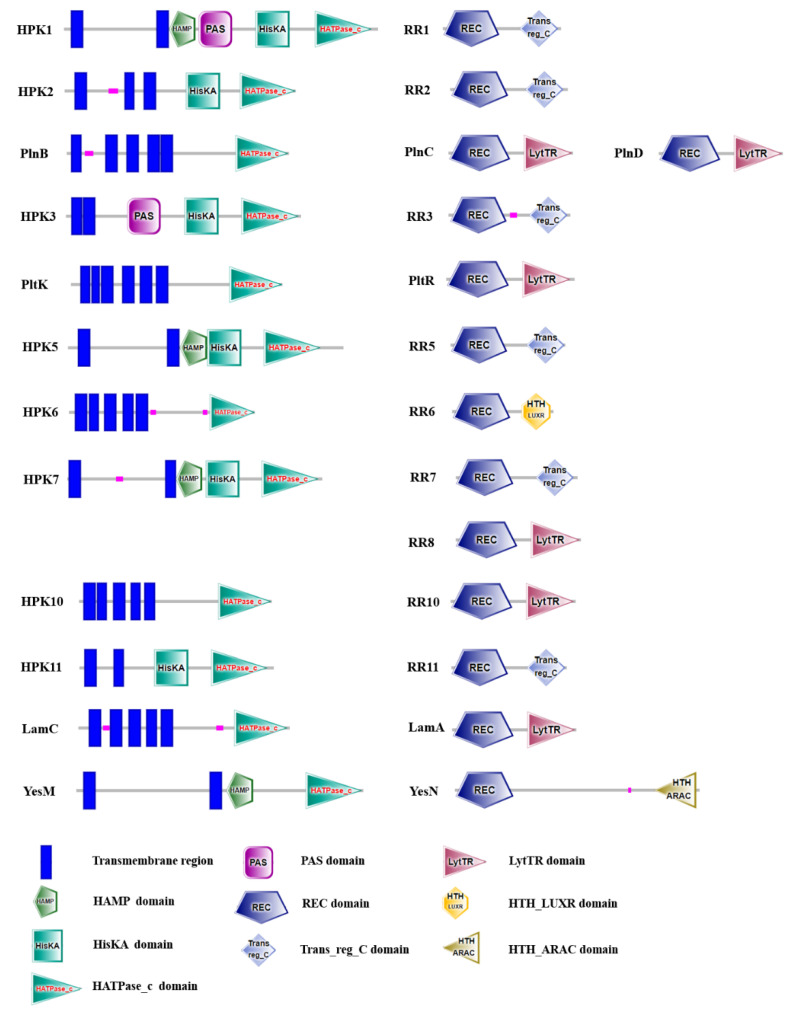
The distribution of TCSs in the *L. plantarum* LP-F1. The TCSs were predicted by SMART domain (http://smart.embl.de/, accessed on 9 December 2021).

Twelve pairs TCSs genes and an orphan RR gene (*rr8*) were found in the genome LP-F1 (Figure 5). The HPK located in the membrane, all HPKs in LP-F1 contain transmembrane regions at their N-terminals. HPK1 and HPK3 possess PAS domain, which is an important signal sensor domain and involved in the perception of various environmental changes, such as oxygen, redox potential, and light [43]. The HisKA domain with a conserved histidine residue is a core domain of HPK, which is responsible for the signal transfer via autophosphorylation.

All RRs in LP-F1 have a conversed REC response regulatory domain and a flexible receptor region. The former contains a phosphor-acceptor site, and is in charge of the accept and transmit of phosphate group. The latter is expected to bind to specific DNA sequences in the upstream regions of target genes. LytTR and Trans-reg_C are major receptor region types. Interestingly, HTH_LUXR and HTH_ARAC domains were found in the RR6 and YesN, respectively.

The genomic analysis indicated that most *L. plantarum* strains contains 10–13 TCSs genes and an orphan RR gene (Supplementary Table S5). TCSs *hpk4/rr4*, *plnBCD*, and *yesNM* are present in 37.88%, 47.20%, and 52.17% of strains with known genome sequences, respectively. The other TCSs genes are common in most strains. Interestingly, there are two copies of *hpk7/rr7* in the strain CXG9, which is very rare in the LAB species. It was speculated that the various TCS distributions in the *L. plantarum* strains might contribute to the different adaptabilities of strains.

### 2.5. Carbohydrate Metabolism Ability Analysis of LP-F1

The LP-F1 genome contains glucose, galactose, sucrose, fructose, lactose, maltose, mannose, and glucose metabolism gene clusters (Supplementary Table S3). Sugar fermentation experiments showed that LP-F1 could utilize the above sugars. No gene clusters related to L-sorbose and L-fucose were found in LP-F1 genome. The results of sugar fermentation showed that LP-F1 could not utilize these two sugars.

Rhamnose metabolism related genes were found in LP-F1 genome (Supplementary Table S3-7). However, the L-rhamnose isomerase gene *rhaA* is a pseudo gene. Sugar fermentation experiments showed that LP-F1 could not ferment rhamnose, which might be related to the inactivity of L-rhamnose isomerase. The results of genomic bioinformatics analysis were consistent with fermentation experiments.

### 2.6. Quantification of the Expressions of Sugar Metabolism Related Genes in LP-F1

In order to clarify the expression changes of sugar metabolism related genes under different carbohydrate fermentation conditions, the expressions of 27 genes were analyzed (Supplementary Table S6).

In the study, FOS contains 10% glucose, fructose, and sucrose mixture. Taking MRS media with 0.2% glucose, 0.2% fructose or 0.2% sucrose as controls, the expressions of genes were analyzed in MRS medium with 2% FOS at 4 h and 12 h (Supplementary Tables S6-1 and S6-2).

Compared with the conditions with 0.2% glucose and with 0.2% fructose, the expressions of FOS and sucrose metabolism genes *sacA*, *sacR1, sacK1*, *pts1BCA*, and *agl2* were up-regulated in MRS medium with 2% FOS at 4 h (Supplementary Table S6-1). Compared with 0.2% sucrose condition, the expressions of FOS and sucrose metabolism genes did not show significant changes.

In addition, glucose metabolism related genes *pts11A*, *pts11BC*, and *pgi* also showed similar expression. Under 2% FOS condition, compared with the condition with 0.2% glucose, the expression of *pgi* was significantly increased, the expressions of *pts11A* and *pts11BC* were not significantly changed. Compared with the conditions with 0.2% fructose and 0.2% sucrose, the expressions of *pts11A* and *pgi* were significantly increased, but *pts11BC* was not obviously changed.

When cultured for 12 h, under 2% FOS condition, compared with 0.2% glucose, 0.2% fructose and 0.2% sucrose conditions, the most genes expressions were increased (Supplementary Table S6-2). The above results showed that under 2% FOS fermentation condition, the change of gene expression was determined by FOS rather than a small amount of glucose, fructose and sucrose.

#### 2.6.1. The Different Fermentation Times

On the whole, with the extension of fermentation time, most sugar metabolism genes expressions were down-regulated (Supplementary Table S6-3).

Compared with those after 4 h fermentation, the expressions of *sacA*, *sacK1*, and *agl4* were significantly up-regulated after 12 h in MRS medium without sugar, and the expression levels of *sacR1* and *agl2* were also increased. In the MRS medium with 0.2% glucose, the expressions of *sacA*, *sacR1*, and *agl2* have no significant change. The expressions of *sacK1*, *pts1BCA*, *pts26BCA*, and *sacR2* were decreased significantly.

In 0.2% fructose, the expression levels of *sacA*, *sacR1*, and *agl4* had no significant change. The expressions of *sacK1*, *agl2*, *pts26BCA*, and *sacR2* were decreased significantly. The expression of *pts1BCA* was increased to 2.179 ± 0.688. In 0.2% sucrose, the expressions of *sacA*, *sacR1*, *sacK1*, *pts1BCA*, *agl2*, and *sacR2* were decreased significantly, while the expression of *pts26BCA* did not obviously change, and the expression of *agl4* was significantly increased.

In 2% FOS, the expressions of *sacA*, *sacR1*, *sacK1*, *pts1BCA*, and *agl2* were decreased significantly, while the expressions of *pts26BCA* and *sacR2* were significantly increased, and the expression of *agl4* did not significantly change. In 2% inulin, except for *agl4* and *pts11BC*, other tested genes expressions were significantly decreased, indicating that the sugar metabolism ability of the strain was weakened at the later stage of fermentation, which was consistent with the results of growth ability test of the strain.

#### 2.6.2. The Effect of Different Sugars on the Expressions of Sugar Metabolism Related Genes under 4 h

Using the MRS medium without sugar as control, the changes of sugar metabolism gene expression under different carbohydrate fermentation conditions for 4 h were analyzed (Supplementary Table S6-4). The addition of glucose, fructose, sucrose, FOS, and inulin promoted significantly most sugar metabolism genes expressions.

When glucose was used as carbon source, the expression of *pts11ABC*, which was responsible for glucose transport, increased significantly, while the expression of *pgi* gene did not significantly change, indicating that the bacteria mainly carried out glucose transport at 4 h of fermentation, and the corresponding transformation did not start immediately. When sucrose, FOS and inulin were used as carbon sources, the expression levels of *pts11ABC*, *pgi,* and *fruK* genes were increased in varying degrees, indicating that glucose metabolism produced by the decomposition of sucrose, FOS, and inulin was active.

When sucrose, FOS or inulin were used as the sole carbon source, except for the genes *pts26BCA* and *agl4* in the *sacPTS26* gene cluster, the expressions of the other genes were up-regulated, especially the genes in *sacPTS1* gene cluster. These results indicated that *sacPTS1* gene cluster plays a leading role in the metabolism of sucrose, FOS and inulin in LP-F1 strain.

When glucose was used as carbon source, there was no significant change in the expressions of *sacA* and *sacR1*, while the expressions of *sacK1*, *pts1BCA*, *agl2*, *pts26BCA, agl4* and *sacR2* were increased. When fructose was used as carbon source, the expression levels of FOS and sucrose metabolism genes *sacA*, *sacR1*, *pts1BCA*, *agl2* and *pts26BCA* did not change significantly, while the expression levels of *sacK1*, *agl4* and *sacR2* were increased.

The expressions of *fruA*, *fruA1*, *pst9D* and *pts10AB* were increased after adding fructose. After adding fructose, sucrose, FOS and inulin, respectively, the expression of *fruK* gene was increased significantly, indicating that the metabolism of fructose produced by the above four kinds of sugars was active. Under the condition of inulin addition, the expressions of *fruA*, *fruA1*, *fruA2*, *fruB* and *pts10AB* genes encoding PTS were increased, especially *fruA*, *fruA1*, *fruA2* and *fruB*. These results suggest that the PTS encoded by the above genes may be involved in the transportation of inulin, which needs further study.

#### 2.6.3. The Effect of Different Sugars on the Expressions of Sugar Metabolism Related Genes under 12 h

Using the MRS medium without sugar as control, the changes of sugar metabolism gene expression under different carbohydrate fermentation conditions for 12 h were analyzed (Supplementary Table S6-5).

Compared with the MRS medium without sugar, the expression levels of glucose PTS transporter PTS11ABC and 6-phosphate-glucose isomerase PGI coding genes had no significant change in glucose condition, indicating that glucose transport and utilization had stabilized after 12 h fermentation. Interestingly, the expression of *pgi* was significantly decreased in the medium supplemented with fructose, sucrose, or inulin, but obviously increased in the medium supplemented with FOS. The above results showed that the metabolism of glucose produced by FOS decomposition was still vigorous after 12 h fermentation.

In the medium containing FOS, the expression of *sacK1* gene was increased, indicating that fructose produced by FOS decomposition was further metabolized. At the same time, the expression of *pts1BCA* was significantly increased, indicating that the transportation of FOS was still active. The expression of *pts26BCA*, another transport system coding gene, was also increased. After 12 h of fermentation, the expressions of most FOS and sucrose metabolism genes were significantly decreased in the inulin medium compared with the sugar free medium, and the expression of *pts1BCA* remained unchanged.

It was speculated that PTS26BCA encoded by *pts26BCA* replaced PTS1BCA in the late stage of fermentation and was responsible for the transport of substrates. The above results showed that FOS and inulin depended on different transport systems after 12 h fermentation. This may be the reason for the difference of metabolic ability of LP-F1 strain to these two sugars.

At 12 h, compared with the medium without sugar, the expressions of most fructose transport and metabolism genes were up-regulated significantly in the FOS medium, except *fruA1*, *fruB* and *mtlR*, indicating that the metabolism of fructose was active. In inulin medium, the expressions of *fruA* and *mtlR* were not significantly changed, and the other genes related to fructose transport and metabolism were significantly decreased, indicating that fructose produced by inulin decomposition was significantly reduced, and fructose metabolism was also significantly decreased.

#### 2.6.4. The Correlation of the Sugar Metabolism Related Genes

**Figure 6:**
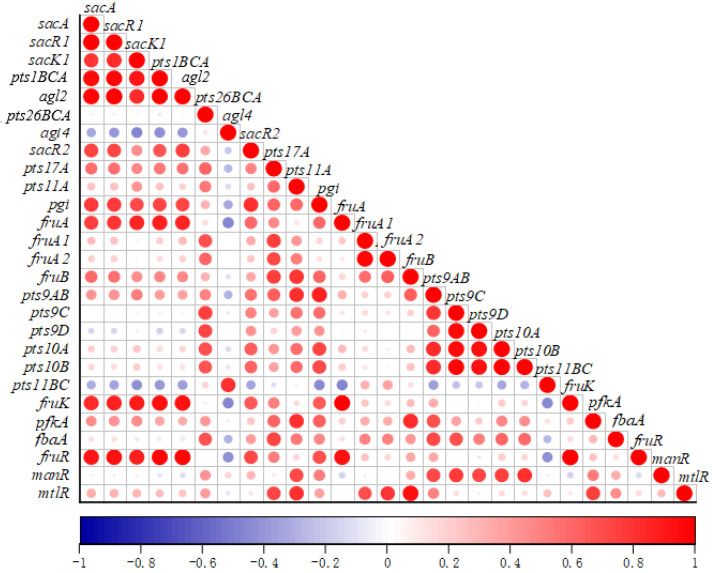
Correlation analysis between the expressions of different sugar metabolism related genes.

According to the expressions of genes in different fermentation conditions, the correlation map of genes expressions was drawn (Figure 6). The results showed that most sugar metabolism related genes were positively correlated with each other, such as *sacA*, *sacR1*, *sacK1*, *pts1BCA*, and *agl2*, while only a few genes were negatively correlated with other genes. For example, *agl4* was negatively correlated with other genes except *pts11BC*.

The genes *sacA*, *sacR1*, *sacK1*, *pts1BCA*, and *agl2*, which are key genes in the FOS metabolism have a high positive correlation with each other, indicating the transport genes, hydrolysis genes and regulatory genes in the pathway of FOS metabolism interact with each other to improve their expressions.

In addition, the expressions of glucose-6-phosphate isomerase encoding gene *pgi*, fructose PTS transporter subunit IIA encoding gene *fruA*, and phosphofructokinase encoding *fruK*, and transcription regulator gene *fruR* were positively correlated with FOS metabolism-related genes. It was speculated that there is a high correlation between genes related to different carbohydrates metabolisms.

### 2.7. Quantification of the Expressions of TCSs Genes in LP-F1

In order to clarify the expression changes of regulatory genes under different carbohydrate fermentation conditions, the expressions of *ccpA* and TCSs genes were analyzed (Supplementary Table S7).

Compared with the MRS media with 0.2% glucose, 0.2% fructose and 0.2% sucrose, the expressions of most genes were up-regulated in the MRS with 2% FOS at fermentation 4 h, for examples, *hpk1*, *rr2*, *plnBCD*, *rr3*, *rr5*, *rr7*, *rr8*, *lamA/lamC*, etc (Supplementary Table S7-1). It is significant that the expressions of *rr6/hpk6* were decreased compared with 0.2% fructose fermentation condition. There was no significant change in the expressions of other genes.

Compared with the MRS media containing 0.2% glucose, 0.2% fructose, and 0.2% sucrose, the expressions of *rr1*, *hpk1*, *hpk3*, and *rr8* were up-regulated in 2% FOS at 12 h (Supplementary Table S7-2). The expressions of *hpk6* and *lamR* were down-regulated. There was no significant change in the expressions of *rr2*, *plnB*, *pltK*, *hpk5*, and *hpk11*. The above results showed that a small amount of glucose, fructose, and sucrose mixed in FOS had no significant effect on the expressions of most genes.

#### 2.7.1. The Fermentation Stages

With the extension of fermentation time, the expression of *ccpA* was significantly up-regulated in the media with fructose, sucrose, and FOS as carbon sources, but was down-regulated in the media without sugar, and glucose, and inulin as carbon sources (Supplementary Table S7-3). The expressions of TCSs genes presented differences under different fermentation conditions with the extension of fermentation time (Supplementary Table S7-3).

In the MRS medium without sugar, after 12 h, compared with 4 h, the expressions of other genes were up-regulated or not significantly changed, except that the *plnC* gene was down-regulated. In MRS medium with 0.2% glucose, the expressions of *lamR* and *yesN* were up-regulated.

In MRS medium with 0.2% fructose, the expressions of *hpk7*, *lamR* and *lamC* were up- regulated. In addition, the expressions of *hpk2*, *plnC*, *plnD*, *hpk3*, *rr7*, and *rr11* were down-regulated. There was no significant change in other genes. In 0.2% sucrose, the expressions of *hpk2/rr2*, *plnBCD*, and *rr3/hpk3* were down-regulated with the extension of fermentation time, while the expressions of *rr5/hpk5*, *rr6/hpk6*, *lamR/lamK*, and *yesN/yesM* were up-regulated.

Interestingly, with the extension of fermentation time, most TCSs genes expressions showed the same trend in 2% FOS and 2% inulin. In 2% FOS, with the extension of fermentation time, the expressions of *hpk* and *rr* in most TCSs showed the same trend, and the expressions of *hpk2/rr2*, *plnBCD*, *rr3/hpk3*, and *rr8* were under-regulated, while the expressions of *pltK/pltR*, *rr6/hpk6*, *lamR/lamK*, and *yesN/yesM* did not change significantly. A few TCSs are specific, such as the expression of *hpk7* was increased and *rr7* decreased. There was no significant change in the expression of *hpk11*, but the expression of *rr11* was decreased.

#### 2.7.2. The Effect of Different Sugars on the Expressions of Genes under 4 h

The expressions of *ccpA* and TCSs genes in different carbohydrate sources were analyzed (Supplementary Table S7-4). Compared with the MRS medium without sugar, the expressions of most genes were significantly up-regulated after the supplement of glucose, fructose, sucrose, FOS, and inulin.

After adding glucose as carbon source, the expressions of most genes, except *ccpA*, *rr1/hpk1*, *hpk5*, *rr7,* and *rr8*, were significantly increased compared with those in the sugar free medium. When fructose was as the sole carbon source, the expressions of *hpk3*, *plnK/plnR*, *rr6/hpk6*, *hpk7*, *lamR/lamK*, *hpk11/rr11*, *lamA*, and *yesN/yesM* were significantly increased, while the expressions of other genes were not significantly changed. Compared with those in sugar free medium, the expressions of *rr2*, *plnBCD*, *rr3*, and *lamA/lamC* were up-regulated in sucrose, but the expressions of other genes were not significantly changed. In contrast, the expression changes of most genes in fructose medium were more significant than that in sucrose medium. It is speculated that the structure of fructose is simpler and easier to be metabolized by the strain, which promotes the rapid growth of the strain and increases the expressions of most genes.

Compared with the sugar free medium, the expressions of *ccpA*, *hpk2/rr2*, *plnBCD*, *rr3/hpk3*, *pltR*, *rr5*, *hpk7/rr7*, *rr8*, *lamR*, *rr11,* and *lamA/lamC* were up-regulated in the medium supplemented with FOS or inulin. It was found that FOS and inulin present similar regulatory effects on the expressions of most TCSs genes.

#### 2.7.3. The Effect of Different Sugars on the Expressions of Genes under 12 h

The expression of *ccpA* was obviously up-regulated at 12 h in the medium containing fructose, sucrose, FOS, and inulin, compared with the medium without sugar (Supplementary Table S7-5).

Compared with the sugar free medium, the expressions of *plnC* and *rr11* in the glucose medium were significantly up-regulated, while the other genes were not obviously changed. In the medium containing fructose, the expressions of *hpk7* and *lamC* were up-regulated by 12.343 ± 1.916 and 2.677 ± 0.194 times, respectively, while the expressions of *hpk2*, *plnC*, *hpk3*, *rr5*, and *rr11* were down-regulated. In a sucrose medium, the expressions of *plnC*, *rr5*, *hpk7*, *rr7*, and *lamC* were up-regulated, and the expression of *hpk7* was increased by 9.297 ± 0.319 times; the expression of *hpk2* was decreased, and the other genes had no significant change.

When FOS was used as the sole carbon source, the expressions of *rr1/hpk1*, *plnC*, *rr5*, *hpk7/rr7*, *rr8*, *rr11*, and *lamC* were increased, and the expression of *hpk1* was up-regulated by 19.956 ± 5.703 times, the expressions of *hpk6*, *lamR*, and *yesN* were decreased, but the expressions of other genes did not change significantly. In the medium containing inulin, the expression of *hpk7* was increased, while the expressions of most other genes were decreased.

### 2.8. Correlation between the Sugar Metabolism Related Genes and Regulatory Genes

**Figure 7:**
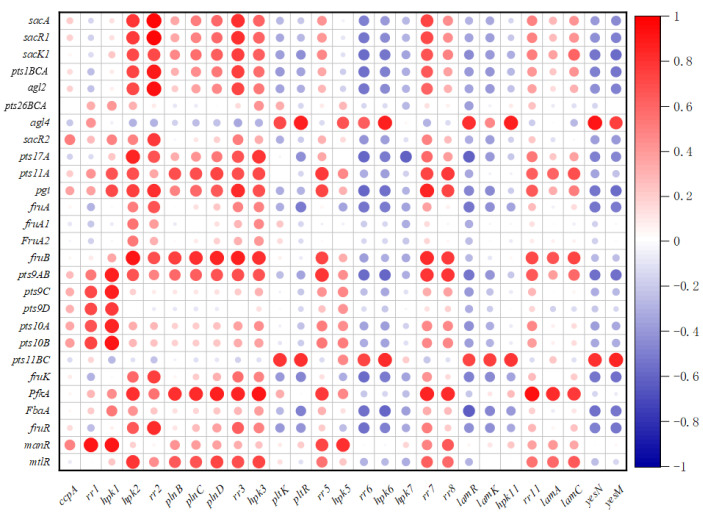
Correlation analysis between the expressions of regulated genes and different sugar metabolism related genes.

According to the expressions of sugar metabolism genes and regulatory genes in different fermentation conditions, the correlation map of different genes was drawn (Figure 7). The *ccpA* has a high positive correlation with regulation related genes *manR* and *sacR2*. In addition, the TCSs genes *rr1/hpk1*, *rr2/hpk2*, *plnBCD*, and *rr3/hpk3* have high positive correlations with most of the sugar metabolism genes, especially with the key genes of FOS transport and metabolism. The regulatory genes *rr6/hpk6*, *hpk7*, *lamR/lamK*, *hpk11*, and *yesN/yesM* were negatively correlated with most of the sugar metabolism genes. The *rr6/hpk6* and *yesN/yesM* were negatively correlated with most other genes except *agl4* and *pts11BC*.

## 3. Discussion

*L. plantarum* is found in various environments and has a strong carbohydrate utilization capability [1,2,3,10,11,12,13]. The *L. plantarum* LP-F1 has a high sugar fermentation ability and shows potential in application. 165 *L. plantarum* strains with known genomic sequences have been analyzed. These results indicated that the species *L. plantarum* has a large number of carbohydrate metabolism genes and presents a strain specificity. Different *L. plantarum* strains showed a plenty of diversities in the sugar metabolism genes profiles. 165 *L. plantarum* strains could be classified into 89 types according to the distribution of carbohydrate metabolism related genes (Supplementary Tables S4-1 and S4-2). The various genes profiles might give strains different sugars fermentation abilities. The result will be beneficial for screening strains with strong carbohydrates metabolism capacities by means of the identification of key sugar utilization genes. Furthermore, it will helpful for the understanding the difference mechanism of different strains with various properties.

As key regulation systems, TCSs play key roles in the growth and adaptability of strains. TCSs genes analysis of 165 *L. plantarum* strains revealed that the species has more TCSs than other LAB, and the distribution of TCSs also showed the strain specificity. The functions of some TCSs in *L. plantarum* and other bacteria have been identified. However, most studies have focused on one specific TCS [23,24,25,26,27,28]. The effects of TCSs distribution in different strains of same species on their properties are rarely studied, although they are interesting topics and will accelerate the screening of strains with excellent characteristics if deeply studied.

Under fermentation, the supplements of glucose, fructose, sucrose, FOS, and inulin promoted the expression of most of the sugar metabolism genes. It was found FOS metabolism key genes *sacA*, *sacR1*, *sacK1*, *pts1BCA*, and *agl2* have high positive correlations with each other, indicating the transport genes, hydrolysis genes and regulatory genes in the FOS metabolism pathway interact with each other to improve their expressions.

In addition, the expressions of fructose metabolism genes (*fruA, fruK, fruR*) and glucose metabolism gene *pgi* were positively correlated with those of FOS metabolism related genes. These results suggested the whole carbohydrate metabolism in bacterium are affected by the types of carbohydrates in the environments, and different carbohydrates utilizations have tight correlations.

Compared with the fermentation condition of sugar free, the expressions of most of the TCSs genes were up-regulated significantly in the media containing sugars, such as *hpk2/rr2*, *plnBCD*, and *rr3/hpk3*. Furthermore, the expression trends of most *hpk* genes and their corresponding *rr* genes in the same TCS were consistent. The correlation analysis between the expressions of regulatory genes (*ccpA* and TCSs genes) and sugar metabolism related genes showed that the expressions of regulatory genes *rr1/hpk1*, *rr2/hpk2*, *plnBCD*, and *rr3/hpk3* were positively correlated with those of most of the sugar metabolism genes, while the expressions of *rr6/hpk6*, *hpk7*, *lamR/lamK*, *hpk11*, and *yesN/yesM* were negatively correlated with those of most of the sugar metabolism genes, suggesting that TCSs might be involved in the regulation of sugar metabolism. The roles of these TCSs in the prebiotics FOS and inulin metabolism require study in the future.

These results indicated that prebiotic utilization of bacteria is a complex process, which is closely related to sugar transport, decomposition, metabolism, and regulatory genes. There remains a lot of work to be performed in order to clarify the complex mechanism of prebiotic metabolism in bacteria.

## 4. Materials and Methods

### 4.1. Bacterial Strain and Growth Conditions

*L. plantarum* strains LP-F1, LP-E1, LP-A4, LP-I5, LP-1, and LP-4 were isolated from fermented milk samples, which were grown in a de Man-Rogosa-Sharpe (MRS) medium under 37 °C and kept in liquid MRS culture with 25% (*w*/*v*) glycerol at −70 °C [29].

### 4.2. Genome Sequencing of the L. plantarum LP-F1

The genome of *L. plantarum* LP-F1 was sequenced using a combination of Pacbio RS and Illumina platforms by LC-BIO (Hangzhou, China). The Illumina data was used to evaluate the complexity of the genome and correct the PacBio long reads. The ABySS (http://www.bcgsc.ca/platform/bioinfo/software/abyss, accessed on 9 December 2021) was used to do genome assembly with multiple-Kmer parameters and got the optimal results of the assembly [44]. Secondly, a canu (https://github.com/marbl/canu, accessed on 9 December 2021) was used to assemble the PacBio corrected long reads. GapCloser software was applied to fill up the remaining local inner gaps and correct the single base polymorphism (https://sourceforge.net/projects/soapdenovo2/files/GapCloser/, accessed on 9 December 2021) [45]. The genome sequence of LP-F1 has been submitted on NCBI and was assigned as CP051192. 15 *L. plantarum* strains genomes available in public databases were used in the construction of phylogenetic tree, including 5-2, ST-III, TMW 1.1478, WCFS1, 16, CAUH2, J26, JDM1, K25, KLDS1.0391, LPL-1, LZ95, LZ206, P-8, and SN35N (Supplementary Table S2).

### 4.3. In Silico Analysis of the Carbohydrate Metabolism Genes of L. plantarum

Carbohydrate-Active enzyme (CAZy) in *L. plantarum* LP-F1 was annotated with dbCAN (http://bcb.unl.edu/dbCAN2/, accessed on 9 December 2021) and CAZy database (http://www.cazy.org/, accessed on 9 December 2021) [46,47]. 165 *L. plantarum* strains genomes available in public databases were used in the carbohydrate metabolism genes analysis (Supplementary Table S2).

### 4.4. Carbohydrate Fermentation Tests

Carbohydrate utilization patterns were evaluated according to the method of Hu et al. (2018) [24]. The 23 carbohydrate fermentation tests were performed to determine the phenotypic variability of strains, including D(+)glucose, D(+)galactose, D(+)fructose, D(+)lactose, D(+)sucrose, D(+)maltose, D(+) mannose, D(+)mannitol, D(-)salicin, D(+)xylose, D(+)ribose, D(+)sorbitol, Galactitol, L(+)-rhamnose, L(-)sorbose, L(-)fucose, L(+)arabinose, D(+)cellobiose, D(+)trehalose, D(+)melibiose, D(+)-melezitose, fructooligosaccharides (FOS), and inulin.

Cells from overnight cultures were pelleted by centrifugation (12,000 g, 5 min) and were then washed and resuspended in peptone broth (peptone 20 g/L, NaCl 5 g/L, K_2_HPO_4_ 0.2 g/L). Then, the cells were cultured in peptone broth with 2% sugar for 24 h at 37 °C, containing 3% BTB-MR (bromo phenol blue 0.2 g, methyl red 0.1 g, 95% ethanol 300 mL, distilled water 200 mL) as the pH indicator. Cell suspension without any sugar was negative control. Sugar fermentation resulted in the change of the color from blue to yellow, and turbidity was increased.

### 4.5. Quantitative Real-Time -PCR

The *L. plantarum* strain LP-F1 was cultivated in MRS media with different carbohydrates (2% FOS, or 2% inulin). The FOS in this study contain 10% other sugars mixture (glucose, fructose, sucrose). Therefore, MRS media with 0.2% glucose, 0.2% fructose, and 0.2% sucrose were used as controls, in order to identify the influences of these sugars. Cells were collected after 4 h (logarithmic phase), and 12 h (stationary phase) cultivation. Total RNA was extracted using Trizol reagent according to the previous method [23]. Total RNA was quantified by measuring the OD_260_ value with SpectraMax iD3 (Molecular Devices, San Jose, CA, USA). Complementary DNA (cDNA) was synthesized by means of the ReverTra Ace qPCR RT Master Mix gDNA Remover (TOYOBO, Osaka, Osaka Prefecture City, Japan).

Twenty-seven genes related to the *L. plantarum* carbohydrate metabolism were selected, focusing FOS metabolism genes (*sacA*, *sacR1*, *sacK1*, *pts1BCA*, *agl2*, *pts26BCA*, *agl4*, and *sacR2*), glucose transport and hydrolysis genes (*pfkA*, *fbaA*, *pts17A*, *pts11A*, *pts11BC*, and *pgi*) and fructose transport and hydrolysis related genes (*fruA*, *fruA1*, *fruA2*, *fruB*, *pts9AB*, *pts9C*, *pts9D*, *pts10A*, *pts10B*, *fruK*, *fruR*, *manR*, and *mtlR*).

Furthermore, the catabolite control protein A (CcpA) gene *ccpA*, as well as twelve pairs of TCSs genes and an orphan *rr* gene (*hpk1/rr1*, *hpk2/rr2*, *hpk3/rr3*, *hpk5/rr5*, *hpk6/rr6*, *hpk7/rr7*, *hpk11/rr11*, *plnB/C/D*, *pltKR*, *lamRK(rr10/hpk10)*, *lamAC*, *yesNM*, and *rr8*) were used in the study. The qPCR primers were designed by Primer3 web [48] (Supplementary Table S8).

Quantitative RT-PCR (qRT-PCR) reactions were performed with Applied Biosystem QuantStudio 5.0 (Thermo Fisher, Waltham, MA, USA) using 2 × SYBR Green qPCR Master Mix (Low Rox) (Bimake, Houston, TX, USA) according to the manufacturer’s instructions. Each reaction was repeated three times. Data were measured using the method of comparative critical threshold (2^−ΔΔCT^) [49]. The 16s rRNA gene was used as a reference gene in all cases. R software was used for correlation analysis.

## 5. Conclusions

In the present study, the distribution of carbohydrate metabolism genes in 165 *L. plantarum* strains with known genomic sequences have been analyzed, indicating different strains present various sugar utilization genes profiles. These results demonstrated that *L. plantarum* is a good material used in the research of carbohydrate metabolism mechanism, and has wide potential applications in prebiotic utilization. FOS, and inulin promoted the expressions of most sugar metabolism genes in *L. plantarum* LP-F1, a potential starter strain with outstanding characteristics. The key genes in FOS metabolism presented high positive correlations. In addition, different carbohydrates utilizations have close relationships. The expressions of regulatory genes (*ccpA* and TCSs genes) and sugar metabolism genes also have tight links. It was speculated that some TCSs might be responsible for the regulation of sugar metabolism in *L. plantarum* LP-F1. 

## Supplementary Materials

The following are available online at https://www.mdpi.com/article/10.3390/ijms222413452/s1.
