# Supplementary material for: The Carbohydrate Metabolism of Lactiplantibacillus plantarum
Source: Int J Mol Sci. 2021 Dec 15;22(24):13452. doi: 10.3390/ijms222413452 (PMC8704671; doi:10.3390/ijms222413452)
Supplement: Supplementary file 1 [file ijms-22-13452-s001.zip › Supplementary Table S1.pdf]

Supplementary Table S1. Carbohydrate utilization of *L. plantarum* strains.

Sugar	Strains					
	LP-F1	LP-E1	LP-A4	LP-I5	LP-1	LP-4
D-Glucose	+++	+++	+++	+++	+++	+++
D-Galactose	+++	+++	++	+++	+++	+++
D-Fructose	+++	+++	+++	+++	+++	+++
D-Lactose	+++	+++	+++	+++	+++	+++
D-Sucrose	+++	+++	+++	+++	+++	+++
D-Maltose	+++	+++	+++	+++	+++	+++
D-Mannose	+++	+++	+++	+++	+++	+++
D-Salicin	+++	+++	+++	+++	+++	+++
D-Xylose	+++	+++	+++	+++	+++	+++
D-Ribose	+++	+++	+++	+++	+++	++
D-Mannitol	+	-	-	+	+	-
D-Sorbitol	+	-	+	+	+	+
Galactitol	-	-	-	-	-	-
L-Arabinose	-	-	++	-	-	++
L-Fucose	-	-	-	-	-	-
L-Rhamnose	-	-	-	-	-	-
L-Sorbose	++	-	++	++	++	-
D-Cellobiose	+++	+++	++	+++	+++	+++
D-Trehalose	+++	+++	+++	+++	+++	+++
D-Melibiose	-	-	-	-	-	-
D-Melezitose	+	-	+	+	++	++
FOS	+++	+++	+++	+	+	+
Inulin	+++	+++	+++	+	+	++

Note: the strain will produce acid substances in sugar utilization, which will make the medium containing bromothymol blue change from blue to yellow. +++, means that the culture color has changed from blue to yellow in 16 h; ++, the medium color changes from blue to yellow in 24-36 h; +, means that the culture color is changed from blue to yellow green in 48 h; -, means the culture color remains blue for 48 h-72 h.
